# Rapid cycling bipolar disorder and atypical anorexia nervosa: changes in drug metabolism

**DOI:** 10.1192/j.eurpsy.2024.753

**Published:** 2024-08-27

**Authors:** J. Teišerskytė, K. Norvainytė

**Affiliations:** ^1^ Lithuanian University of Health Sciences; ^2^Department of Psychiatry, Kaunas Hospital of the Lithuanian University of Health Sciences, Kaunas, Lithuania

## Abstract

**Introduction:**

Bipolar disorder (BD) is a complex mental illness described by recurrent episodes of mania and depression. One subtype of the illness is rapid cycling BD, characterized by experiencing four or more extreme mood swings within a year. Diagnosing and treating BD can be complicated by comorbid conditions, such as atypical anorexia nervosa (AAN), marked by disordered eating and disturbing weight-related thoughts.

**Objectives:**

To discuss the diagnosis and treatment plan of a patient with rapid cycling BD, who experienced adverse effects from prescribed medication and later was diagnosed with comorbid AAN.

**Methods:**

We present a case of a 21 year-old man initially presenting with anxiety, low mood, and obsessive weight-related thoughts, ultimately diagnosed with major depression and mixed anxiety disorder.

**Results:**

21 year-old man was diagnosed with major depression and mixed anxiety disorder, initially treated with mirtazapine and fluoxetine (limited success), later attempting escitalopram and bupropion combination (partial remission). After 2 years the patient discontinued the treatment due to feeling “euphoric”, subsequently experiencing depression and manic episodes – the initial diagnosis was rapid cycling BD. The treatment was changed to sodium valproate (up to 1500 mg/day) and aripiprazole (up to 10 mg/day), however extremely rare adverse medication effects (nosebleeds, diarrhea with blood admixture, “high-frequency sounds”) were reported. Throughout valproate treatment, the patient experienced persistent diarrhea. During hospitalization for treatment adjustment lithium carbonate was introduced at a starting dose of 900 mg/day, maintaining blood lithium levels between 0.4 mmol/l and 0.49 mmol/l. Later the dose was adjusted and a therapeutic lithium blood level was reached with 1575 mg/day of lithium carbonate. Additionally, risperidone was prescribed, however, the patient experienced an uncommon adverse reaction – nasal congestion. Subsequently, amisulpride was introduced, which provoked severe anxiety and fear, resulting in medication discontinuation. During the latest outpatient visit, fluoxetine was added to the treatment due to observed depressive symptoms. Throughout the treatment, the patient episodically intermittently starved, had persistent distressing thoughts about weight and was diagnosed with AAN. While planning further treatment it was hypothesized that comorbid AAN might affect drug metabolism and the patient was referred to a specialized inpatient facility for eating disorder management.

**Conclusions:**

This case report highlights the complexity of psychiatric disorders and the importance of monitoring and adjusting treatment based on patient response and side effects. Additionally, it emphasizes comorbid conditions significance in influencing the primary disorder’s dynamics as well as the metabolism and effectiveness of psychiatric medications.

**Disclosure of Interest:**

None Declared

